# Optimizing recruitment strategies for healthy older adults in prevention clinical trials: A scoping review

**DOI:** 10.1017/cts.2025.10205

**Published:** 2025-12-19

**Authors:** Lauren Barbish, Melissa M. Crane, Brittney S. Lange-Maia, Tarisha Washington, Santosh Basapur, Raj C. Shah

**Affiliations:** 1 Rush Medical College of Rush University Medical Center, Chicago, USA; 2 Department of Family and Preventive Medicine, Rush University Medical Center, Chicago, IL, USA; 3 Rush Alzheimer’s Disease Center, Rush University Medical Center, Chicago, IL, USA

**Keywords:** Older adults (75+), clinical trial recruitment, healthy aging, belonging framework, scoping review

## Abstract

Older adults aged 75 and older (75+) represent the fastest-growing demographic in the USA yet remain underrepresented in prevention-focused clinical research. This scoping review evaluated recruitment strategies used in healthy aging clinical trials targeting this population, with particular attention to technology-enabled and belonging-focused approaches.

A PubMed search initially identified only four US-based studies focused on adults aged 75+. To broaden the scope and enrich the analysis, additional studies involving adults aged 65+ and those with pre-existing conditions were included, yielding a total of 23 relevant studies. Recruitment strategies were analyzed using the Design for Belonging framework to assess how inclusion and engagement were fostered.

Findings revealed that adults aged 75+ preferred traditional methods – targeted mailings, phone calls, and in-person outreach – due to barriers related to digital access and usability. In contrast, adults aged 65+ showed greater receptivity to digital tools such as electronic health records, social media, and web-based enrollment. Community engagement and culturally tailored materials are effective across all age groups. However, few studies addressed later-stage engagement strategies like advocacy and trust repair.

These results underscore the importance of tailoring recruitment strategies to aging subgroups, combining personalized outreach with inclusive design to enhance equity and retention in clinical research.

## Introduction

The aging population in the USA is expanding rapidly, particularly among adults born between 1946 and 1964. By 2034, older adults are projected to outnumber children for the first time in U.S. history, with individuals aged 65+ expected to represent 21% of the population by 2030, up from 15% today [1]. Among these, adults aged 75 and older represent the fastest-growing segment, with this group expanding at a rate of 25.1% [2]. Life expectancy within this age group continues to rise, with males at age 75 living an additional 10.6 years and females 12.5 years on average [3]. These demographic trends highlight an urgent need to engage older adults – particularly those aged 75+ – in prevention-focused clinical research to address their unique health needs. Prevention trials are defined as clinical studies designed to prevent disease onset in individuals without prior history or to reduce recurrence among those at risk, using interventions such as medications, vaccines, lifestyle modifications, or behavioral approaches [4]. Prevention trials are important because they target the maintenance of health, delay of disease onset, and preservation of function in older adults; outcomes that are central to healthy aging yet insufficiently represented in the current evidence base, particularly among adults aged 75 years and older.

However, recruiting adults in this age group remains a persistent challenge. Barriers include strict eligibility criteria, exclusion of non-English speakers and individuals with comorbid conditions, technology-related difficulties such as limited digital literacy, and logistical issues like transportation and time commitment [5]. Although prior systematic reviews have examined recruitment barriers for adults aged 65+, few have focused specifically on the 75+ population or explored the role of technology-based strategies in improving enrollment [6].

At the same time, the rise of digital health tools and virtual engagement presents new opportunities to enhance recruitment efforts. While loneliness and social isolation remain significant concerns for older adults – with chronic loneliness predicting increased healthcare utilization [7] – technology has been shown to foster social connection. Three out of four Americans aged 65 and older report using technologies such as cell phones, the internet, and email, with many using these tools to seek information and maintain contact with family and friends [8]. These trends raise important questions about how technology can be leveraged not only to reach older adults but also to foster the sense of belonging that may drive participation and retention in clinical research.

Fostering belonging is a critical yet often overlooked component of successful recruitment. Research demonstrates that individuals are more likely to engage with initiatives where they feel valued and included [9]. Although this concept has been well established in academic settings – where technology-enhanced courses improve students’ persistence and sense of belonging [10] – its application to clinical trial recruitment, particularly among older adults, remains underexplored. The Design for Belonging framework (see Table 1) offers a valuable lens for understanding how inclusion and connection influence engagement across fields, including healthcare [11]. Rooted in human-centered design principles, this framework identifies key “Moments of Belonging” – such as *invitation, entering, joining, contributing, growing,* and building *community* – that shape how individuals experience connection, safety, and value within a given space or process. Originally applied in educational and organizational contexts to foster equity and participation, Design for Belonging emphasizes intentional strategies to cultivate environments where people feel welcomed, respected, and empowered to engage fully. Applying this framework raises key questions: How does technology impact the sense of belonging for older research participants? Can digital solutions enhance engagement without introducing additional barriers?

**Table 1.** Definitions of key moments of belonging with study recruitment application examples

Key moment of belonging	Definition	Application (Example)
Invitation	Establishing a welcoming environment through personalized outreach.	Personalized calls or letters from trusted senior center staff
Enter	Facilitating initial access and lowering barriers to participation.	Home visits, transportation support, or simplified consent
Join	Encouraging individuals to become active members of the research community.	Small group intros to the team with a clear purpose for their involvement
Contribute	Providing opportunities for participants to share their perspectives and expertise.	Asking for feedback on study materials or visit experience
Dissent	Creating a space for constructive feedback without exclusion.	Hosting seminars or webinars to openly discuss feelings about the study and improvements
Grow	Supporting continuous engagement and development within the community.	Sharing study updates or offering health education sessions
Make demands	Allowing participants to advocate for their needs and improvements in the process.	Adapting study procedures based on requests
Repair	Addressing barriers and fostering relationship restoration when trust has been compromised.	Personal follow-up after missed visits or acknowledging prior mistrust
Dance	Celebrating achievements and fostering joy in participation.	Thank-you cards, small gifts, or community appreciation events
Community	Sustaining long-term engagement and cultural integration.	Partnering with senior centers and aging services for ongoing connection

The COVID-19 pandemic accelerated technology adoption among older adults, with telehealth use among individuals aged 70 and older rising from 4.6% pre-pandemic to 21.1% [12]. Furthermore, 74% of adults aged 75 and older now use more than one digital health device, and nearly 70% rely on multiple digital solutions to support their healthcare needs [13]. As these trends continue, understanding how technology can be integrated into recruitment strategies for the 75+ population is increasingly important.

The purpose of this scoping review is to examine existing recruitment strategies for enrolling adults aged 75+ into prevention and translational research studies. This review focuses on how current methodologies incorporate technology and belonging-focused approaches, identifies key barriers and facilitators, and highlights opportunities for improving engagement. Through this analysis, we aim to inform future recruitment efforts and advance participation of this growing demographic in clinical research.

## Methods

To appropriately evaluate current literature on recruiting older, healthy adults to prevention trials, a scoping review was performed to generate insights into current field evidence on recruitment methods for older adults. This review sought to identify and map key characteristics and concepts within the existing literature. Therefore, a scoping review was chosen over a systematic review, as the purpose was not to evaluate intervention effectiveness but rather to explore the breadth of knowledge in the field. Scoping reviews are particularly well suited for identifying key concepts, mapping existing evidence, and clarifying knowledge gaps – reasons that align with the indications outlined by Munn *et al*. [14]. The Preferred Reporting Items for Systematic Reviews and Meta-Analyses extension for Scoping Reviews (PRISMA-ScR) Checklist was used to ensure compliance and objective selection of articles for review [15]. The protocol for this scoping review was not registered before publication.

### Scoping review search strategy

A review of the literature included articles published from November 2009-2024. The database searched was PubMed. We queried PubMed with the following search terms:

1. (recruit*[ti]) AND (clinical trial*) AND (75 years or older*)

The analysis began by focusing on recruitment strategies for healthy older adults aged 75+ in prevention trials. Initially, studies specifically targeting this age group were prioritized. Due to the limited number of articles, the inclusion criteria were relaxed to include both healthy and older adults with a previous condition in prevention trials. After this inclusion criteria were added, the articles identified were then categorized into two main groups: healthy aging studies and condition-specific studies. Due to the continued limited number of articles in the age 75+ group, the criteria were broadened to include adults aged 65+. This adjustment allowed further subdivision of the articles into healthy aging studies and condition-specific studies for a comprehensive evaluation.

### Eligibility, study selection, and data extraction

A scoping review was conducted using PubMed as the primary database, with Covidence used to support study screening and data management [16]. PubMed IDs were converted into RIS files for upload into Covidence, where references were imported and automatically screened for duplicates.

The title and abstract screening were performed according to predefined inclusion and exclusion criteria. The review specifically sought studies that targeted adults aged 75+, given the underrepresentation of this population in clinical research. Eligible studies included prevention trials, healthy aging populations, and individuals with a previous condition. All recruitment methods were considered, including both traditional and technological interventions. Outcomes of interest included identification of successful recruitment strategies, reasons older adults declined participation, and recommendations for improving enrollment approaches. Studies were limited to those published within the last 20 years.

Exclusion criteria included studies conducted outside of the USA, those published more than 20 years ago, or those focused on young adult populations. Non-U.S. studies were excluded to ensure the review focused on recruitment strategies relevant to the U.S., where health systems, technology adoption, and patterns of aging differ from those in other countries. These contextual differences influence both recruitment and which adults reach older ages, particularly beyond 75 years. Limiting the scope to U.S.-based studies also ensured the findings could inform national prevention efforts and support evidence used by the U.S. Preventive Services Task Force. Treatment trials involving participants with a previous condition and studies targeting adults younger than 65 were also excluded. Narrative reviews, scoping reviews, and systematic reviews were excluded. Screening decisions were made by LB and discussed with RS. Consistent with the objectives of a scoping review, a formal quality assessment and risk of bias evaluation were not conducted.

Data extraction followed a structured template designed to capture key characteristics of each study. General study information was collected, including the citation, participant age range, total number of initial respondents, total number of participants enrolled, health status of the population (healthy aging vs. condition-specific), primary health area studied, and study design. The data template also distinguished between actual enrollment studies and hypothetical studies that assessed participant interest. This contextual information supported interpretation of recruitment strategies across studies.

Primary recruitment strategies and detailed descriptions of recruitment processes were extracted, with attention to reported challenges, success indicators, and participant-level barriers. Facilitators of recruitment and effective strategies used to address these barriers were also noted. Key findings were summarized, including innovative methods, recruitment outcomes, and recommendations for future research. Additionally, the role of inclusive design was evaluated through the application of the *Design for Belonging* framework, assessing how belonging-related principles may have influenced recruitment outcomes.

During data extraction, the primary recruitment strategies were categorized into nine distinct groups based on shared characteristics and common outreach approaches across the included studies. These categories, their abbreviations, and descriptions are outlined in Supplementary Table 1.

*The Design for Belonging* toolkit (see Table 1) was utilized to analyze the articles, identifying recruitment themes from a sense of belonging perspective to formulate suggestions and highlight current strategies for improving recruitment among older adults [11]. The frequency of each key moment of belonging was calculated separately for the 65+ and 75+ groups and reported in the results.

Finally, data were stratified by participant health status (healthy aging vs. condition-specific) and by age group (65+ and 75+) to identify trends, assess recruitment effectiveness, and inform future strategies (see Supplementary Table 2).

### Qualitative analysis plan

In this scoping review, a qualitative analysis was conducted to identify strategies within each article that align with the *Design for Belonging* framework. Key “Moments of Belonging” were identified and mapped across the reviewed literature. These moments represent pivotal interactions where individuals may experience either inclusion or exclusion, offering opportunities for strategic design interventions to enhance belonging.

A deductive thematic analysis was employed in this review. Deductive thematic analysis is a structured qualitative research method that applies predefined themes or concepts drawn from existing theories or prior research to qualitative data. This top-down approach ensures that data analysis is guided by established frameworks, allowing for a systematic categorization of findings [17]. In this review, deductive thematic analysis was used to categorize strategies within each study according to the key “Moments of Belonging” (see Table 1).

Each article was systematically reviewed to extract data relevant to thematic categories. Strategies were then assessed for their effectiveness in fostering belonging and inclusivity among older adults. The frequency of key moments of belonging mentioned in the articles was calculated for both the healthy aging and disease progression groups. Comparisons across studies helped identify best practices, recurring barriers, and gaps in current recruitment approaches.

## Results

### Study selection and characteristics

**Figure 1.**
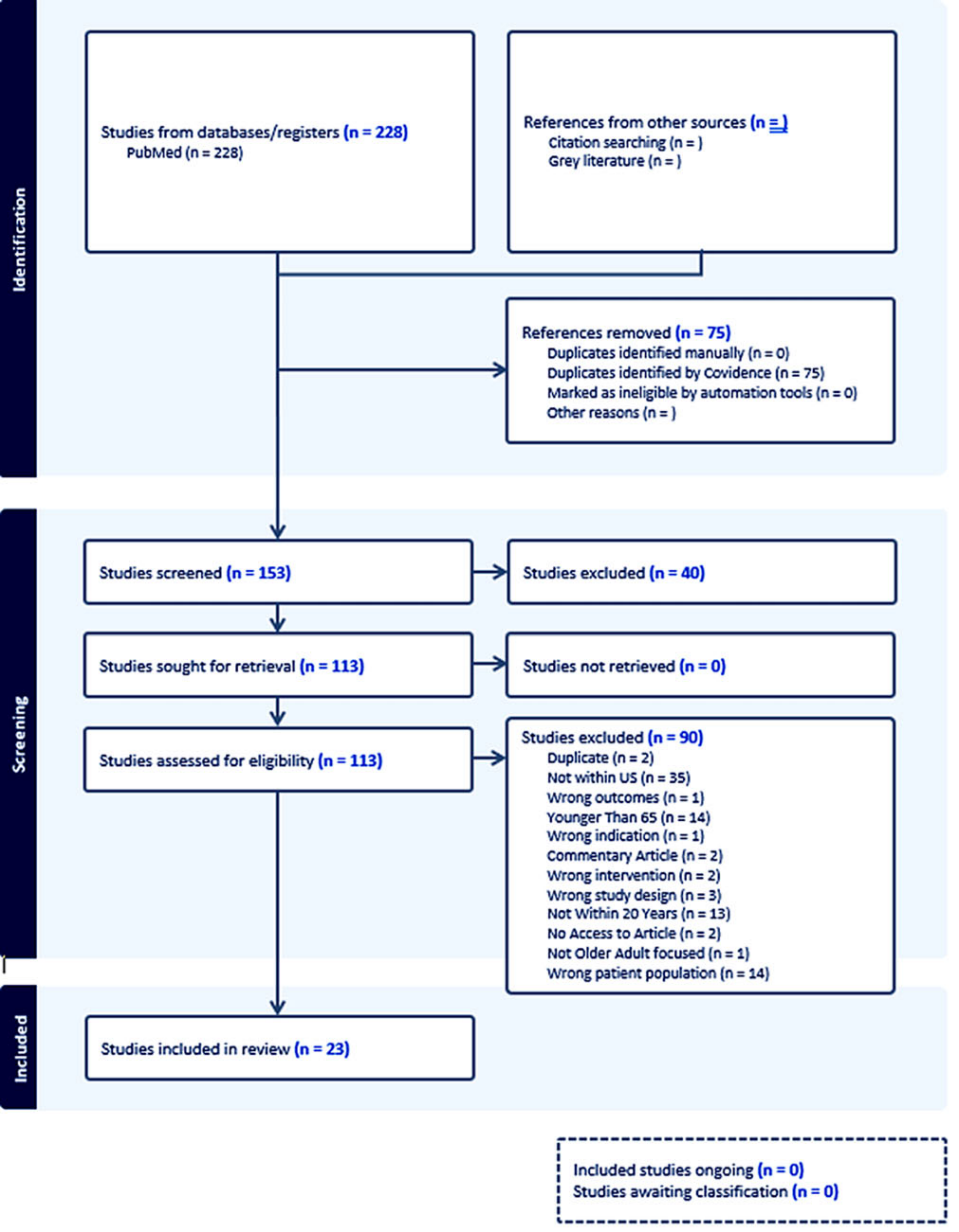
Prisma flow diagram.

Database searches from PubMed retrieved a total of 228 papers, which yielded about 153 unique papers after de-duplication. In screening the titles and abstracts, 40 were excluded because of irrelevant populations, study type, setting, or location of study. The initial search filtered for articles between 2004 and 2024 (20 years), but there were no articles present about this subject until 2009. After further screening, investigators reviewed 113 papers of which 23 articles were identified to have fulfilled the inclusion criteria. Most articles were excluded due to conduct outside of the USA (*n* = 35) (see Figure 1).

In the overall scoping review, 4 out of 23 articles specifically mentioned adults aged 75+, while 19 out of 23 referenced adults aged 65+. Of the 23 studies included in this review, 3 were classified as *hypothetical trials*, in which participants were asked about their stated willingness or likelihood to enroll in a clinical trial, but no actual enrollment occurred. These studies were used to explore attitudes toward participation and identify potential recruitment facilitators or barriers. All 3 of these hypothetical studies focused on adults aged 65+. In contrast, the remaining 20 studies involved *actual enrollment* of participants into prevention trials, providing more concrete data on recruitment outcomes and strategy effectiveness.

**Figure 2.**
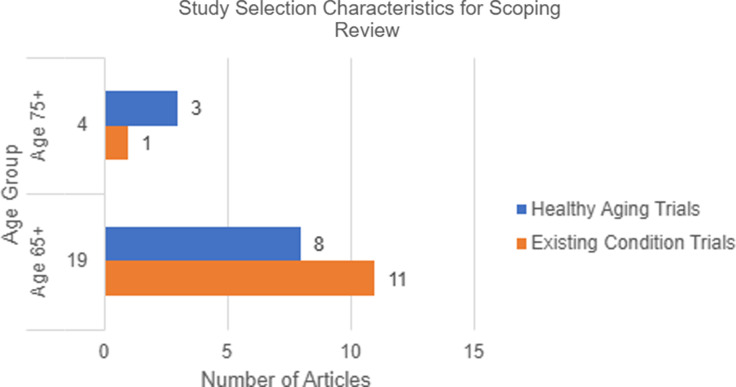
Study characteristic comparison for scoping review.

When examining recruitment for healthy aging and disease prevention trials, 11 out of the 23 articles focused on enrolling healthy older adults in prevention clinical trials. Among these, 3 articles targeted individuals aged 75+ [18–20], while 8 articles focused on those aged 65+ [21–28]. In contrast, 12 articles focused on recruiting participants with an existing condition or disease to a prevention trial. Of these, only one article specifically mentioned adults aged 75+ [29], whereas 11 discussed recruitment of adults aged 65+ [30–40] (see Figure 2).

The prevention studies examined recruitment strategies for older adults aged 65+ and 75+, targeting both healthy individuals and those with pre-existing conditions, defined as chronic or previously diagnosed medical conditions present prior to enrollment in the study. For healthy participants, studies focused on enrolling individuals without major diagnoses such as heart disease, stroke, or dementia, with outcomes centered around measures like disability-free survival. For participants with pre-existing conditions, the studies recruited individuals with clearly defined diagnoses – such as stroke survivors participating in trials aimed at preventing recurrent strokes. Pre-existing conditions and primary health areas studied across the studies included cognitive impairment (Alzheimer’s disease, mild cognitive impairment), cardiovascular and kidney disease, medication adherence challenges, mobility impairment and functional decline, physical function and exercise needs, and cancer survivorship and wellness.

### Recruitment strategies for adults aged 75+

Of the four studies enrolling healthy participants over the age of 75, none evaluated addressed the use of clinic-based technologies in recruitment efforts. One study compared recruitment outcomes using Facebook pages and DialMyCall, a mass notification system that delivers automated phone calls and text messages. The study reported a 0% recruitment rate through Facebook pages, whereas phone-based outreach demonstrated a slightly higher recruitment rate (0.6%); however, both methods yielded recruitment rates under 1% [18]. Targeted mailing has also been identified as an effective recruitment approach. One study found that sending study invitations to zip codes with a high proportion of the target population improved recruitment outcomes [19].

While not specifically focused on technology implementation, the Healthy Black Elders Program demonstrated that educational materials could play a crucial role in advancing recruitment among adults aged 75+. The program reported the highest proportion of screened participants, suggesting that culturally tailored, family-influenced approaches may enhance recruitment within this population [19]. In the I-CONECT trial [19], researchers found that many older Black participants preferred to consult with family members before providing consent, highlighting the value of incorporating trusted relatives into the recruitment process. This practice aligns with cultural norms emphasizing strong intergenerational bonds and collective decision-making, which helped facilitate clearer communication of study details and improved willingness to participate. Additionally, educational interventions may help improve participant engagement and diversity. Providing potential participants with educational materials about the significance of clinical trial participation has been shown to enhance understanding and motivation, thereby increasing recruitment diversity [20]. These findings underscore the importance of using personalized, community-driven, and education-based recruitment strategies to improve participation rates among older adults in healthy aging research.

In contrast, one study examined various recruitment methods targeting older adults, particularly those aged 75+ in disease prevention studies [29]. Strategies included site-based recruitment at locations treating older adults, outreach at senior centers, targeted mailing lists, mass mailings, brochures, posters, referrals, hospital and clinic chart reviews, engagement with healthcare providers, peer referrals, and the use of mass media. Among these methods, clinic-based technologies played a critical role in identifying and recruiting older participants. Electronic medical record (EMR) searches, computerized participant records systems, and automated clinic referral alerts allowed researchers to efficiently filter potential participants based on age, medical history, and inclusion criteria, such as hypertension and chronic kidney disease. Some clinical sites used computerized participant records systems to internally generate recruitment lists, reducing reliance on external vendors, while automated EMR referral alerts enabled healthcare providers to flag and refer eligible older adults during routine visits [29].

In addition to clinic-based technologies, communication technologies such as targeted mass mailings and mass media outreach significantly contributed to recruitment efforts. Mass mailings proved to be highly effective, accounting for 39.8% of recruited individuals aged 75+, with third-party vendor lists, voter registration records, and other publicly available mailing lists being the most successful sources [29]. These digitally optimized mass mailings allowed for age-targeted recruitment while maintaining cost efficiency. However, web-based recruitment strategies had minimal impact, as older adults were less likely to engage with online platforms for clinical trial enrollment.

Non-technology-based recruitment strategies were also essential in successfully enrolling older participants. Direct referrals from healthcare providers, peer referrals, and community-based engagement at senior centers and hospitals played a key role in increasing enrollment. Many older adults were more likely to participate in the study when recommended by a trusted clinician, reinforcing the importance of human-driven recruitment approaches. Additionally, increasing the number of recruitment sites and strategically allocating resources toward locations with high concentrations of older adults further enhanced the likelihood of meeting enrollment goals [29]. Ultimately, a combination of clinic-based technologies, effective communication strategies, and direct interpersonal engagement proved to be the most effective approach for recruiting older adults aged 75+ to clinical trials (Table 2).

**Table 2.** Summary of findings for older adults aged 75+

Age Group: 75+	Key findings
Healthy adults [18–20]	Phone calls and text messaging are more effective than social media (0% recruitment via Facebook).^18^ Targeted mailings to specific zip codes improve recruitment.^19^ Community-based programs (e.g., Healthy Black Elders Program) increase participation. ^19^ Educational interventions enhance diversity and engagement.^19,20^ Direct outreach methods (phone calls, text messages, mailings) preferred.^18^ Trusted community programs and partnerships with retirement communities improve recruitment.^20^ Geographic targeting, family involvement, and educational initiatives enhance participation. ^19^
Pre-existing conditions [29]	Mass mailings (39.8% of recruits) most effective.^29^ Other strategies: site-based recruitment, community outreach, provider referrals, media use.^29^ Expanding recruitment sites improves outcomes.^29^ Targeting specific subgroups increases participation.^29^

### Recruitment strategies for adults aged 65+

Recruitment strategies were explored for older adults aged 65+ into healthy aging studies. Recent studies have explored the role of technology in recruitment, particularly through electronic health record (EHR) portals and email. Remote recruitment via email was effective in enrolling older adults, especially when using patient portals [23,24]. However, both studies highlighted challenges in reaching diverse populations, as technology use is often lower in certain demographic groups.

Beyond EHR portals and email, targeted mailings have been shown to be an effective recruitment method for older adult populations. One study reported that targeted mailings resulted in a 2–6% response rate, compared to just 1% for non-targeted mailings [21]. Typically, study brochures or personalized letters were mailed to households with age-eligible residents, using commercial databases or voter registration lists. Targeted mailings to health plan members have also been identified as successful [22].

Other non-digital recruitment strategies focus on leveraging social networks and community trust. One study found that the most common recruitment sources were internal site methods (45.4%) and earned media (39.9%). Internal sources, such as referrals from other studies and community outreach, are particularly effective in engaging diverse communities by leveraging trust and social networks [25]. This is further emphasized by findings that in-person outreach and screening, conducted by diverse staff at familiar community venues, increased participation [26]. Partnering with local health departments that routinely conduct health education and screenings may also increase recruitment success, as it builds credibility within the community. Community-based education programs can serve as a bridge to research participation, with 52% higher enrollment from 2018 to 2019 in lifestyle prevention trials among older African Americans after incorporating an Aging with Grace (AWG) curriculum presentation within African American-serving organizations [27]. Additionally, an “Ambassador Program” in which current participants recruited others, or promoting the study through local media, such as a Sunday newspaper feature, effectively increased recruitment number [28].

Clinical-based technology, communication technology, and non-technological interventions were evaluated in disease prevention studies. Health plan-based recruitment strategies have demonstrated efficacy in clinical settings. One study highlighted the value of administrative claims data from a Health Plan Research Network in pragmatic clinical trial recruitment [38]. The integration of multiple outreach methods, including direct mail, email, and telephone, resulted in a reported response rate of 9–10%. However, email recruitment was limited by a higher rate of undeliverable addresses compared to postal mail, reinforcing the advantage of direct mailing for this population. Expanding the number of study sites further enhanced recruitment by increasing the reach of direct mail campaigns and broadening participant diversity based on study site locations [36]. Establishing relationships with healthcare institutions and community organizations was also associated with increased trust and improved recruitment outcomes.

Technology-based interventions outside of clinical settings have been proposed to address accessibility barriers, offering greater flexibility and routine engagement for individuals with transportation difficulties [40]. Web-based study platforms allowed potential participants to access information at their convenience without requiring study staff involvement [34]. Direct interpersonal engagement with study staff fostered trust and increased willingness to participate in high-commitment studies [31]. Participants who received a caring vignette describing the study were more than twice as likely to enroll compared to those receiving an uncaring vignette, underscoring the importance of interpersonal rapport in recruitment efforts.

Alternative recruitment strategies that do not rely on technology have also been explored. A predominant finding across studies is the effectiveness of direct mail campaigns targeted by age and geographic location, which consistently yielded the highest recruitment rates [30,37,40]. One study evaluated an ambassador referral program in which past participants were invited to promote the study to others. While direct mailing remained the most effective method (34.7% of enrolled participants), ambassador referrals accounted for 15.3% of recruitment and were the most cost-efficient strategy [35]. Similarly, another study found that word-of-mouth referrals and community partnerships were the most effective recruitment methods, contributing to over 49% of participant enrollment [32]. Additional findings reported that featuring studies in local newspapers facilitated recruitment of diverse populations. Moreover, 78.62% of participants were recruited through face-to-face interactions at community centers and events, while 83% were enrolled through community hospital senior services programs [33]. These findings suggest that recruitment sources may influence participant characteristics, with community hospital programs being more likely to enroll individuals with comorbidities, whereas studies targeting healthy aging populations less frequently utilized hospital-based providers for recruitment. Additional factors influencing recruitment include flexibility, convenience, and transportation barriers. Overall, the evidence suggests that a multifaceted approach incorporating direct mail, community partnerships, word-of-mouth referrals, and personalized engagement strategies is most effective for optimizing recruitment in clinical research (Table 3).

**Table 3.** Summary of findings for older adults aged 65+

Age Group: 65+	Key findings
Healthy adults [21–28]	Targeted mailings (2-6% response rate) outperform non-targeted mailings (1%).[21] Email and EHR portals work but face accessibility challenges.[23,24,28] Community referrals and outreach (e.g., health departments) improve recruitment.[26] Education programs increase enrollment among African Americans (52% boost).[27] Peer recruitment (“Ambassador Program”) and local media coverage (e.g., newspaper features) are effective.[27,28] Mailed brochures and personalized letters yield the highest recruitment rates.[21,22] Digital recruitment shows promise but requires trust-building efforts.[23-25] Community engagement, including local media and healthcare professionals, is critical.[25-28]
Pre-existing conditions [30–40]	Direct mail campaigns yield the highest recruitment rates.[30,36-40] Health plan-based recruitment (EHR, claims data) effective but email less reliable.[30,34,38] Peer referrals and community partnerships boost recruitment (49% from word-of-mouth).[30,32,36-40] Personal engagement (e.g., caring study descriptions) increases willingness to participate.[31] Web-based platforms improve accessibility but need better inclusion strategies.[34] Trust and pre-existing relationships with health institutions are crucial.[30,36] Community partnerships, local media features, and transportation assistance improve recruitment.[32,33,35]

### Key moments of belonging

**Figure 3.**
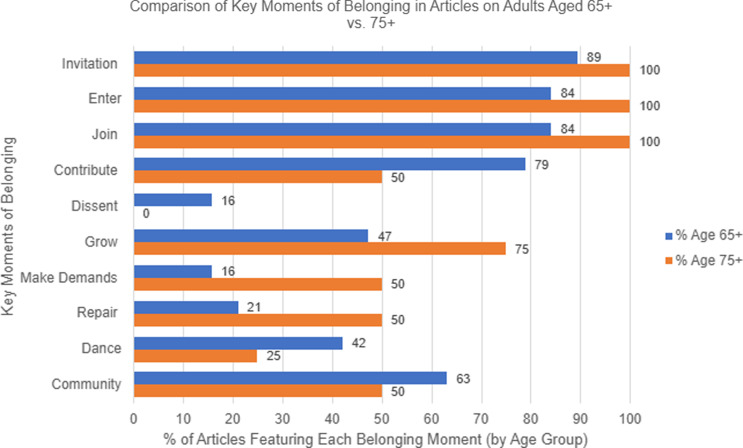
Analysis of key moments of belonging across articles for older adults aged 65+ and 75+.

The thematic deductive analysis revealed key moments of belonging across different age groups of older adults. Among adults aged 75+, the most consistently present key moments of belonging were *invitation*, *enter*, and *join*, which appeared in all four articles analyzed. These moments primarily focused on initial interactions with study staff. For example, *invitation* was demonstrated by using traditional methods like calling and texting, which proved more effective than social media outreach for the PREVENTABLE clinical trial [18]. The *enter* moment was reflected in strategies such as posting flyers in Continuing Care Retirement Communities (CCRCs), soliciting wellness or activity directors to assist in recruitment [20], and expanding the number of study sites to increase participant reach [29]. Lastly, the *join* moment was captured through the intentional design of advertisements, outreach strategies, and study materials aimed at both educating potential participants and engaging influential individuals in their lives [19]. *Grow* was present in three articles, while *contribute, make demands, repair*, and *community* appeared in two. *Dance* was identified in one article, and *dissent* was absent across all four studies (see Figure 3).

In the broader analysis of 19 articles focusing on adults aged 65+, *invitation* was the most frequent theme (17/19), followed closely by *enter* and *join* (16/19). *Contribute* was present in 15 articles, while *community* appeared in 12. *Grow* (9/19) and *dance* (8/19) were less prevalent but still notable. *Repair* appeared in four articles, whereas *make demands* and *dissent* were the least common, each occurring in only three articles (see Figure 3). Several studies repaired trust through direct actions such as offering personal follow-up phone calls when participants did not respond [33], acknowledging historical mistrust by openly discussing past research abuses in culturally tailored Alzheimer’s education programs like *Aging with Grace* [27], and employing consistent, culturally concordant staff to maintain relationships with socially isolated Black adults [19]. Others humanized the research process by using caring recruitment behaviors, such as answering all participant questions rather than distributing a flyer [31], or by adding videos of staff and former participants to recruitment websites to increase transparency [34]. These findings suggest that narratives of belonging for older adults emphasize invitation and participation but less frequently highlight themes of dissent or advocacy, particularly among the oldest cohort (75+).

## Discussion

The aim of this scoping review was to evaluate and optimize recruitment strategies for prevention studies targeting healthy older adults aged 75+. Due to the limited number of studies focused exclusively on this age group, the scope was expanded to include studies involving adults aged 65+ and those with existing health conditions. In total, the review assessed 11 articles on healthy aging prevention trials and 12 on disease progression trials, with a range of strategies evaluated across both. These findings reinforce the importance of tailoring recruitment methods to the specific needs of older adult subgroups.

As summarized in Table 4, many strategies were effective across both the 65+ and 75+ populations. Traditional methods such as targeted mailings, personalized phone calls or texts, in-person outreach, community-based education, clinical referrals, ambassador programs, culturally tailored materials, mass media, and recruitment through churches or community groups consistently yielded strong results. These approaches often involved geographic targeting and leveraged established relationships – aligning with the most common key moments of belonging (e.g., *invitation, enter, join*). Future work should explore how recruitment strategies can be designed not only to foster belonging but also to integrate technology in meaningful, accessible ways.

**Table 4.** Comparison of effectiveness of recruitment methods for 65+ and 75+

Category	Recruitment strategy	Best for 65+	Best for 75+	Less effective / needs adaptation
Mass mailings / direct mail	Targeted mailings	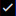	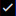	
Digital outreach	EHR portals / email invitations	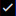		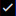 (75+: technology access barriers)
	Web-based enrollment platforms	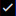		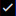 (75+: requires tech support)
	Electronic consent / digital forms	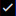		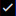 (may deter 75+ due to usability concerns)
	Social media (e.g., Facebook recruitment)	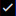		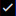 (0% success in 75+ group)
Phone/text-based outreach	Phone calls / text messages (e.g., DialMyCalls)		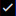	
In-person / community outreach	In-person outreach (e.g., senior centers, events)	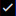	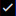	
	Community-based education programs	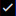	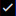	
	Recruitment via Church / religious / community groups	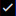	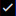	
Health system–based recruitment	Trusted clinician referrals	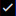	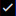	
Media & advertisements	Use of mass media (TV, newspaper, radio)	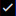	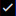	
Referral-based strategies	Peer recruitment / ambassador programs	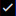		
Study-specific custom Approaches	Involving family members in decision-making		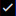	
	Geographic targeting (e.g., zip code-based outreach)	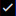	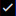	
	Culturally tailored educational materials	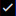	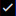	

Findings revealed that older adults – especially those aged 75+ – strongly preferred traditional recruitment methods over digital outreach. The most successful strategies centered on community partnerships, geographic targeting, and involving family members in decision-making. In-person interactions were particularly important in fostering belonging and motivating participation, indicating that a sense of personal connection and trust remains essential for this age group.

In contrast, the 65+ group demonstrated greater openness to both traditional and digital methods. Social media, web-based enrollment, electronic consent, and EHR portal invitations were more effective for this younger subset of older adults, reflecting a generational shift toward greater digital literacy and comfort. To maximize recruitment, hybrid strategies that blend relationship-based outreach with emerging technology may be most effective for this population.

Technology-heavy approaches – including EHR invitations, email, social media, online enrollment, and e-consent – were largely ineffective or required significant adaptation for adults aged 75+. Common barriers included low access to technology, usability concerns, and a need for assistance. Providing ongoing tech support, simplifying interfaces, and offering training may help reduce these barriers and improve the feasibility of digital recruitment for this group.

This scoping review intentionally applied the *Design for Belonging* framework to examine recruitment strategies through a relational and inclusion-centered lens, moving beyond the typical focus on logistical or structural barriers. Key moments identified in this framework – such as *invitation*, *enter*, *join*, *contribute*, *grow*, and *community –* emphasize the importance of making participants feel welcomed and part of something meaningful. For instance, in-person recruitment events like health fairs or professional endorsement sessions serve as structured “joining” mechanisms that build community and foster a sense of shared purpose. These strategies not only increase recruitment rates but also support long-term trust, which is critical for sustained engagement in clinical trials.

However, analysis of these moments revealed significant gaps in later-stage engagement, including limited opportunities for *dissent*, *advocacy*, and *repair*. Most strategies focused on initial recruitment phases (e.g., *invitation and joining*) without offering ways for older adults to voice concerns (*Dissent*), advocate for their needs (*Make Demands*), or rebuild trust when issues arise (*Repair*). Addressing these later moments could enhance both recruitment and retention. Establishing forums or town halls where older adults can express concerns may help strengthen trust and promote sustained participation. For those with mobility or transportation barriers, web-based forums supported by ongoing tech assistance may offer a viable solution. For example, You et al emphasized the importance of providing 24/7 tech support in remote trials [40], while Yu et al. found that conversational engagement via the I-CONECT intervention increased comfort with technology among older adults [41]. Further research has shown that studies conducted in the home environment, with user-friendly hardware and assistance, can reduce barriers to participation [41]. Continued exploration of how to introduce adults aged 75+ to technology will be essential to closing these accessibility gaps.

This review’s strengths lie in its comprehensive assessment of recruitment strategies across various contexts and age groups. However, several limitations must be acknowledged. Few studies focused exclusively on adults aged 75+, which led to a broadening of the inclusion criteria. Still, the use of technology-based recruitment strategies among adults aged 65+ may offer a preview of future trends. As this group ages into the 75+ population, their growing comfort with digital tools could enhance the effectiveness of technology-driven recruitment methods over time. Additionally, the heterogeneity in study designs and sample sizes may limit generalizability, and only one research database was used. Expanding database searches in future reviews could help capture additional relevant studies. This review also did not extract or analyze the inclusion and exclusion criteria of the included trials, which may have influenced recruitment outcomes by shaping the characteristics and accessibility of the study populations. Future work should examine how eligibility requirements affect recruitment feasibility, particularly in prevention trials involving older adults. Additionally, this review considered both recruitment response and yield, though future work should examine these outcomes separately to better understand the factors influencing each.

There are several key gaps in the literature that warrant future investigation. More research is needed on recruitment strategies tailored specifically to adults aged 75+, especially in prevention and disease progression trials. Although digital recruitment methods show promise, many older adults still prefer traditional approaches. Studies like the PREVENTABLE trial, which combines EHR-based identification with mail, phone, and in-person outreach, may offer valuable insights into the feasibility of mixed-method strategies for the oldest age groups [42]. Long-term retention strategies and methods for maintaining engagement and trust over time also remain underexplored.

This review aligns with prior scoping review research, including findings from Rodriguez [43], which reported that older adults are more responsive to traditional recruitment strategies. However, it expands upon earlier work by highlighting the distinct needs of those aged 75+, a group often underrepresented in recruitment studies. While some digital strategies may be suitable for younger older adults, traditional methods remain the most trusted and effective for the oldest participants.

Practically, these findings support the implementation of community-based engagement strategies in clinical and research settings. Clinics can collaborate with retirement communities, senior centers, and local health providers to host in-person sessions where potential participants can ask questions and learn about ongoing studies. Personalized follow-up via mail, phone, or text can reinforce these efforts and convey a sense of personal invitation. Involving family members and leveraging trusted messengers – such as community leaders or local media – can further boost participation rates and strengthen trust.

## Conclusion

This review highlights that effective recruitment of adults aged 75+ hinges on traditional, trust-based strategies – such as personalized outreach and community partnerships – while digital tools require substantial adaptation to meet their needs. Incorporating belonging-centered approaches and addressing later-stage engagement gaps will be critical for enhancing both recruitment and long-term participation in clinical trials.

## Supporting information

10.1017/cts.2025.10205.sm001 Barbish et al. supplementary material Barbish et al. supplementary material

## Supplementary material

The supplementary material for this article can be found at https://doi.org/10.1017/cts.2025.10205
