# Supplementary material for: Optimizing recruitment strategies for healthy older adults in prevention clinical trials: A scoping review
Source: J Clin Transl Sci. 2025 Dec 19;9(1):e282. doi: 10.1017/cts.2025.10205 (PMC12780804; doi:10.1017/cts.2025.10205)
Supplement: Barbish et al. supplementary material [file S2059866125102057sup001.docx]

**Supplementary Table 1.** Recruitment Strategy Categories with Abbreviations

| **Abbreviation** | **Category** | **Description** |
| --- | --- | --- |
| **MM** | Mass Mailings | - Broad distribution of identical recruitment materials to demographically defined audiences (e.g., ZIP code, age group). |
| **DM** | Direct Mail | - Targeted mailings to specific individuals identified through health records, prior programs, or previous study participation. |
| **DO** | Digital Outreach | - Use of digital platforms such as email campaigns, patient portals, social media, and study-specific websites. |
| **PTO** | Phone/Text-Based Outreach | - Recruitment through phone calls, text messages, or automated messaging systems. |
| **ICO** | In-Person/Community Outreach | - Face-to-face recruitment at community events, health fairs, senior centers, educational talks, or through outreach ambassadors. |
| **HSR** | Health System-Based Recruitment | - Use of healthcare system resources, including electronic medical record alerts, patient registries, and provider referrals. |
| **MA** | Media & Advertisements | - Paid or earned media exposure through television, radio, newspapers, newsletters, press releases, or other mass media channels. |
| **RS** | Referral-Based Strategies | - Recruitment through physician referrals, past participants, community organizations, or advocacy groups (e.g., Alzheimer’s Association). |
| **SSA** | Study-Specific/Custom Approaches | - Tailored recruitment methods designed to meet unique study needs, including localized plans, ambassador programs, or culturally specific outreach. |

**Supplementary Table 2.** Description of Individual Studies Included in Scoping Review

| **First Author, Year** | **Age Range** | **Total # of Initial Respondents** | **Total # of Participants Enrolled** | **Condition or Healthy** | **Primary Health Area Studied** | **Hypothetical vs. Actual Study** | **Study design** | **Primary Recruitment Strategies** | **High Yield Strategies** | **Key Moments of Belonging (see Table 1)** |
| --- | --- | --- | --- | --- | --- | --- | --- | --- | --- | --- |
| Wright 2015 | ≥75 | 319 | 219 | Healthy | Physical Function | Actual | Cross sectional study | - ICO - DM - ICO | - Flyer + wellness director = highest yield - Random sampling + follow-up letters = lower yield, reduced volunteer bias | *Invitation, Enter, Join, Grow, Make Demands, Repair, Community* |
| Struble 2023 | ≥75 | 1139 | 186 | Healthy | Cognitive Health | Actual | RCT | - RS - MM - ICO - RS - DO + MA | - Community-based Healthy Black Elders program = highest screening yield - Targeted zip code mailings = 2nd highest yield - Family involvement in consent = improved recruitment | *Invitation, Enter, Join, Contribute, Grow, Make Demands, Repair, Dance, Community* |
| Clinton 2024 | ≥75 | 1075 | 3 | Healthy | Cognitive Health | Actual | Case report | - DO - PTO | - DialMyCall (0.6%) > Facebook (0%) for PREVENTABLE trial recruitment | *Invitation, Enter, Join* |
| Ramsey 2016 | ≥75 | 14,692 | 9361 | Condition | Cardiovascular | Actual | RCT | - SSA - ICO - MM - HSR + RS - MA | - Mass mailings = 39.8% (highest yield) - Brochures/posters = 30% - Referrals (staff, providers, friends) = 23.1% - Media (TV, radio, newspaper, web, toll-free) = 16.6% | *Invitation, Enter, Join, Contribute, Grow* |
| Santoyo-Olsson 2011 | ≥65 | 5,114 | 238 | Healthy | Metabolic Health | Actual | RCT | - SSA - ICO - RS - DO - MA | - Person-to-person + partner outreach = 60%+ enrollment - Tabling = 39 randomized participants - Screenings + diabetes education = 90% interest post-risk disclosure - Trusted orgs + bilingual materials = 78% minority, 32% Spanish-speaking, 31.9% aged 65+ | *Invitation, Enter, Join, Contribute, Grow, Dance, Community* |
| Raman 2021 | ≥65 | N/A | 5945 | Healthy | Cognitive Health | Actual | Cross sectional study | - HSR - RS - MA | - Internal site sources (45.4%): other studies, community outreach, clinic referrals, site mailings/brochures, physician referrals, local registries - Earned media (39.9%): 52.5% from local coverage, 38.4% from national | *Invitation, Enter, Join, Dance, Community* |
| Plante 2020 | ≥65 | 116 | 12 | Healthy | Fall Prevention | Actual | RCT | - HSR | - Patient portal messages matched email marketing response rates, had minimal opt-outs, and improved with longer, content-rich messaging and timed delivery - Specific, targeted messaging reduced screen failures; first study to use portal recruitment without physician consent | *Invitation, Enter, Join, Contribute* |
| Ng 2023 | ≥65 | 7012 | 5899 | Healthy | Cardiovascular | Actual | Descriptive, observational | - DO | - Email recruitment effective for older adults in the right context - Higher response when emails come from patient’s current healthcare system | *Invitation, Enter, Join, Contribute, Grow, Community* |
| Martinson 2010 | ≥65 | 6451 | 1049 | Healthy | Physical Function | Actual | RCT | - MM - DM - HSR - RS | - Direct mailings = majority of enrollees (n=824) - Self-referral = ~25% of enrollees (n=225) | *Dissent, Grow, Make Demands* |
| Marsh 2013 | ≥65 | 14,812 | 1635 | Healthy | Physical Function | Actual | RCT | - SSA - MA - HSR | - Study brochures and personalized letters mailed to age-eligible households (found via commercial databases and voter registration lists) = 59.4% of contacts, 59.5% of randomizations - Mass mailings of brochures and letters = efficient recruitment method | *Invitation, Enter, Join, Contribute* |
| Stout 2020 | ≥65 | 546 | 97 | Healthy | Cognitive Health | Actual | Case report | - ICO - MA - DO - RS | - Front-page newspaper article = 394 responses (highest yield) - Word-of-mouth/snowball sampling = 66 responses; moderate success across groups - Print media attracted both non-Hispanic White (NHW) and African American (AA) participants - Social media yielded mostly NHW participants | *Invitation, Enter, Join, Contribute, Grow, Community* |
| Shadyab 2021 | ≥65 | 992 | 296 | Condition | Cognitive Health | Actual | RCT | - HSR - ICO - MA - MM - PTO | - Identified appropriate audiences using age- and geo-targeted mailings; framed messaging around brain health instead of disease - Focus groups used to refine recruitment content and impact - Multiple outreach strategies implemented in parallel for broader reach | *Invitation, Enter, Join, Contribute, Grow, Community* |
| Sciamanna 2021 | ≥65 | 6818 | 1139 | Condition | Fall Prevention | Actual | Qualitative research | - DM - HSR - PTO - MA - ICO | - Mailed screening letters = primary recruitment source - Best yield: EHR-identified patients with prior fragility fracture (65:1 mail-to-enroll ratio) - Higher response from Penn State Health patients (trusted relationships) - Poorest yield: purchased list from marketing company (Lorton Data) - Mailed letters = scalable to recruitment goals - Multiple diverse sites increased demographic representation | *Contribute, Dance, Community* |
| Nkimbeng 2020 | ≥65 | 1713 | 300 | Condition | Physical Function | Actual | RCT | - MM - RS - SSA - MA | - Community outreach via Baltimore city programs + community-based organizations yielded 49.7% of participants | *Invitation, Enter, Join, Contribute, Grow, Dance, Community* |
| McHenry 2015 | ≥65 | 1394 | 276 | Condition | Cardiovascular/Medication Use | Actual | RCT | - ICO - PTO - DM | - use of social marketing principles and forming solid relationships with community-based organizations - combination of face-to-face contact with the provision of service, such as blood pressure checks, was successful | *Invitation, Enter, Join, Contribute, Dissent, Grow, Make Demands, Repair, Dance, Community* |
| Greimel 2022 | ≥65 | 396 | 96 | Condition | Cognitive Health | Actual | RCT | - MA - DM - RS - ICO - DO | - Word-of-mouth referrals + community partnerships = 49%+ of participants (most effective strategies) | *Invitation, Enter, Join, Contribute* |
| You 2023 | ≥65 | 334 | 32 | Condition | Fall Prevention | Actual | RCT | - MM - DM - MA | - targeted mailings to specific area codes were successful in recruiting over 100 eligible diverse older adults - Mass mailings followed by opt-in telephone screening surveys included in the letters were successful | *Invitation, Enter, Join, Contribute* |
| Snyder 2009 | ≥65 | 2156 | 641 | Condition | Cancer | Actual | RCT | - HSR - RS - DO | - 26,000 letters mailed + 2,000+ calls made to reach accrual target - Waiver for direct mailing improved accrual and expanded reach to rural and minority older adults across North Carolina | *Invitation, Enter, Join, Contribute* |
| Shi 2020 | ≥65 | 1549 | 355 | Condition | Cardiovascular | Actual | Descriptive, Observational | - PTO - ICO - DO - DM - HSR | - Phase 1 (mail/email outreach): 0.7% response rate, 26.7% of responders enrolled - Phase 2 (brochure): 1.9% response rate, 12.7% of responders enrolled - Brochure drove more portal visits; mail/email yielded higher enrollment rate | *Invitation, Enter, Join, Grow, Community* |
| Cauley 2019 | ≥65 | 5424 | 289 | Condition | Physical Function | Actual | RCT | - MM - ICO - HSR - MA - RS - PTO - SSA - DM | - Direct mail (brochure/postcard) = 83% of participant contacts (most common and effective) - Highest yields from direct mail, electronic medical record–based outreach, and advertisements | *Invitation, Join, Contribute, Dance, Community* |
| Shaw 2022 | ≥65 | 66 | N/A | Healthy | Cognitive Health | Hypothetical | Descriptive, Observational | - SSA | - Targeted, community-based recruitment enhanced enrollment of older African Americans in Alzheimer's and related dementia trials - Culturally tailored prevention curriculum effectively recruited African American participants - In-person education and discussion increased community engagement - Collaborating with African American organizations enabled use of familiar, trusted spaces for outreach | *Invitation, Enter, Join, Contribute, Dissent, Grow, Make Demands, Repair, Dance, Community* |
| Miller 2021 | ≥65 | 2605 (Exp 1), ,374 web (Exp 2) | N/A | Condition | Fall Prevention | Hypothetical | RCT | - MM - DO - ICO - MA - RS+DO | - Original landing page (no infographics/videos) = highest online interest form response - Study website generated strong response yield among web users | *Invitation, Enter, Repair* |
| Gamboa 2023 | ≥65 | 120 | N/A | Condition | Cognitive Health | Hypothetical | Cross sectional study | - DM | - Caring, personalized recruiter behaviors (Swanson’s Theory of Caring) = 5-fold increase in enrollment; equally effective for African American and Non-Latinx White older adults - Proactive, relationship-based recruitment outperformed reactive methods; direct engagement and trust-building were key | *Invitation, Enter, Join, Contribute Repair, Dance, Community* |

**Abbreviations:** RCT, Randomized Controlled Trial; MM, Mass Mailings; DM, Direct Mail; DO, Digital Outreach; PTO, Phone/Text-Based Outreach; ICO, In-Person/Community Outreach; HSR, Health System-Based Recruitment; MA, Media & Advertisements; RS, Referral-Based Strategies; SSA, Study-Specific/Custom Approaches (see Supplemental Table 1).
